# A review of RCTs in four medical journals to assess the use of imputation to overcome missing data in quality of life outcomes

**DOI:** 10.1186/1745-6215-9-51

**Published:** 2008-08-11

**Authors:** Shona Fielding, Graeme Maclennan, Jonathan A Cook, Craig R Ramsay

**Affiliations:** 1 Section of Population Health, Division of Applied Health Sciences, University of Aberdeen, Polwarth Building, Foresterhill, Aberdeen, AB25 2ZD, UK; 2 Health Services Research Unit (HSRU), Division of Applied Health Sciences, University of Aberdeen, UK

## Abstract

**Background:**

Randomised controlled trials (RCTs) are perceived as the gold-standard method for evaluating healthcare interventions, and increasingly include quality of life (QoL) measures. The observed results are susceptible to bias if a substantial proportion of outcome data are missing. The review aimed to determine whether imputation was used to deal with missing QoL outcomes.

**Methods:**

A random selection of 285 RCTs published during 2005/6 in the British Medical Journal, Lancet, New England Journal of Medicine and Journal of American Medical Association were identified.

**Results:**

QoL outcomes were reported in 61 (21%) trials. Six (10%) reported having no missing data, 20 (33%) reported ≤ 10% missing, eleven (18%) 11%–20% missing, and eleven (18%) reported >20% missing. Missingness was unclear in 13 (21%). Missing data were imputed in 19 (31%) of the 61 trials. Imputation was part of the primary analysis in 13 trials, but a sensitivity analysis in six. Last value carried forward was used in 12 trials and multiple imputation in two. Following imputation, the most common analysis method was analysis of covariance (10 trials).

**Conclusion:**

The majority of studies did not impute missing data and carried out a complete-case analysis. For those studies that did impute missing data, researchers tended to prefer simpler methods of imputation, despite more sophisticated methods being available.

## Background

Randomised controlled trials (RCTs) are perceived as the gold-standard evaluation method for evidence based medicine. Increasingly quality of life (QoL) outcomes are measured in clinical trials of new treatments, as this is becoming an important factor in decision making. Common QoL instruments include the generic questionnaires such as SF12/SF36 [1] or the shorter EuroQoL EQ5D [2]. Where appropriate, disease specific questionnaires may also be included. Often these outcomes are collected via postal questionnaires and consequently subject to a certain amount of missing data. This can lead to a potential bias in the results if the missing data are not adequately handled. Often assumptions are made about the missingness, which may or may not be appropriate.

Missing QoL data can be very informative in its own right. QoL data is a subjective patient reported outcome which perhaps makes it more sensitive than other outcomes to missing data assumptions. The data may be missing because a patient is not well enough to complete questionnaires or take part in interviews. Disregarding those patients without QoL information is likely to bias the results, affect trial conclusions and ultimately clinical practice. Therefore, it is important to make use of as much data as possible from as many patients as possible.

There are many ways researchers deal with missing data. For example complete-case analysis, available case analysis, joint modelling, pattern mixture models and the focus in this paper – imputation. Simple imputation is a process whereby a reasonable alternative value is substituted for one that is missing. Common procedures include last value carried forward (LVCF), regression and mean imputation. Both regression and mean imputation can be undertaken at a population level or specific to an individual. A more sophisticated approach is multiple imputation, which imputes several values, creating several datasets. Each dataset is analysed separately and the results are combined [3]. The current literature recommends this over simple imputation [4,5] but researchers may overlook its advantages as simple imputation is easier to implement.

A brief overview of some methods for missing data is provided and is by no means exhaustive. The aim of this review was to identify the imputation methods (if any) currently adopted by researchers analyzing and reporting the results of clinical trials with regard to the quality of life outcomes. This review was undertaken as part of a larger project and hence restricted to QoL outcomes in RCTs. Issues surrounding missing data will be similar in other studies and for other types of outcomes.

### Missing data theory

#### Missingness mechanism

In any study there will be reasons why data are missing, which may or may not be related to the outcome of interest. The main mechanisms for missing data are described in detail, in Little and Rubin [3]. In simple terms, missing completely at random (MCAR) is where the missingness is unrelated to outcome (past, present or future). Missing at random (MAR) can be assumed if the missingness is related to observed data (outcome or other collected data). Finally missing not at random (MNAR) occurs if missingness is associated with unobserved data. There are two broad types of missingness pattern: monotone and non-monotone (intermittent). Monotone (or terminal) missing data occurs when responses are provided at every assessment until a given time and thereafter missing. Non-monotone occurs if missing data occurs in between observed assessments.

#### Analysis methods

One way to deal with missing data is to ignore it completely and undertake a complete-case analysis. This method is the easiest to perform, but has the potential to remove a large portion of the patients from the analysis. This method has two major disadvantages in that it reduces the sample size (and thus power of study) and additionally may produce biased results unless the data are MCAR, which may be an unrealistic assumption. An alternative is available case analysis, and in a longitudinal setting one example is repeated measures ANOVA, which assumes MAR data. All patients who provide at least one outcome measure of a series can be included. This increases the size of sample for the analysis. Specific treatment differences at a given time point can be calculated using the data available. Other options for model-based methods include joint modelling, selection models, or pattern mixture models [6,7], the details of which out with the scope of this paper.

#### Imputation

Under simple imputation a single alternative value is substituted for a missing value. This is followed by a complete-case analysis strategy on the augmented dataset.  Examples of simple imputation strategies are last value carried forward (LVCF), mean imputation (calculated on observed data), hotdeck (random selection from those observed) and regression (using other variables in the dataset) [8,9]. Multiple imputation is a process where several values (e.g. five) are imputed to create multiple datasets [3]. The chosen analysis method is performed on each dataset and the results combined. The advantages are that it has the ability to perform complete-case analysis but reflects the uncertainty of the imputed value. In addition, the accuracy of the standard errors is improved. With the advances in computer software, multiple imputation can easily be carried out. In the statistical software package SAS use *PROC MI *(to carry out imputation) and *PROC MIANALYZE *(to combine results) or within STATA use the *ICE *command. Theoretically, multiple imputation methods can handle MAR and MNAR. In the case of MNAR, the dropout process can be modelled and incorporated into the MI procedure. However, this model cannot be verified (since required data are missing) and the analysis is quite sensitive to the dropout model. Therefore, most of the MI procedures require the MAR assumption [10]. Several approaches can be used for MI including regression, propensity scoring or Monte Carlo Markov Chain (MCMC) imputation [11,12]. The choice between these depends on your variable type (continuous, ordinal or nominal) and pattern of missingness (monotone or non-monotone).

## Methods

A PubMed search was carried out to identify RCTs published during 2005 and 2006 in the four leading medical journals: BMJ, Journal of the American Medical Association (JAMA), Lancet and New England Medical Journal (NEMJ). A random selection of a half was sought from the articles identified. The focus of the review was 'imputation to deal with missing QoL outcomes', therefore only those studies which included QoL outcomes were considered for further investigation.

### Data extraction

Data extraction was a two-stage process. During the first stage information on each RCT included: outcome and type (primary and QoL); single or repeated endpoints; amount of missing information; was imputation used; was the mechanism of missingness discussed. Data extraction of each study was undertaken by a single researcher, with queries resolved by consulting a second reviewer. To assess consistency between reviewers' two papers were doubly abstracted by all reviewers. No inconsistencies were shown.

Once those articles with QoL outcomes were identified, a second more detailed data abstraction was undertaken. This obtained more detailed information with regard to missing data and how it was dealt with. Information collected at stage two included: Study details – type of study, study setting, treatments, number of participants and patient demographics (age, gender etc); Proportion of missing outcome data in each treatment arm; Is analysis complete case analysis or do they account for missing data either with modelling or imputation for the primary endpoint; Analysis method used for primary analysis – e.g. repeated measures ANOVA; Imputation details – what method, effect on analysis (sensitivity analysis); Is missing data mechanism identified.

## Results

The literature search described above produced 568 articles for potential inclusion. Following a process of random selection, 285 (50%) articles reporting RCTs during 2005 and 2006 were identified. A QoL outcome (primary or secondary) was reported in 61 papers (21%) and form the basis of this review. The majority of these were published in the BMJ (n = 27, 44%) with 17 (28%) published in NEJM, ten (16%) in the Lancet and the remaining seven (12%) in JAMA.

### Description of missing data

Table 1 describes the proportion of missing data split between studies which did and did not employ imputation. Of the 42 studies that did not perform imputation techniques, six did not provide enough information to determine the proportion of missing cases. Of the remaining, 36, 16 studies had less than 10% missing data in the primary QoL endpoint. This is in contrast to the 5 of 19 studies that used an imputation method. Of the remaining 14 studies which used imputation, the proportion of missing data was unclear for six.

**Table 1:** Proportion of missing data within the 61 studies with QoL outcomes

	Number of Studies		
Proportion of missing data	No imputation	Imputation	Total
None	6	0	6
< 10%	16	5	21
11–20%	6	5	11
>20%	8	3	11
Unclear	6	6	12

Total	**42**	**19**	**61**

Current CONSORT guidelines [13] for the reporting of RCTs require authors to provide a flow diagram of participants in the trial. This should detail the withdrawals and reasons for withdrawal. The majority of trials (n = 50, 82%) contained within this review did provide the flow diagram and reasons for missingness such as withdrawal, death or other medical problems. However, there was no detailed discussion of these reasons and the impact they may have had on the analysis and subsequent results. In only one study, the mechanism of missingness was discussed and was found to be non-ignorable [14].

### QoL measures used

The range of QoL measures used in trials covered by this review was considerable. Nine different generic QoL measures were utilised including: General Health Questionnaire (GHQ) – seven trials; SF12/SF36 – 14 trials; WHOQOL – one trial; Global assessment of functioning (GAF) – one trial; EuroQoL EQ5D – five trials. Due to the differing disease areas the sample of trials covered, there were a large number of disease specific measures used. Examples included: Asthma QoL score, dermatology life index, rhinoconjunctivitis QoL measure, irritable bowel disease questionnaire (IBDQ), Alzheimer's Disease Assessment Scale and the Oswestry pain score. The post treatment follow up using these QoL measures ranged from one to five assessments, with the majority being collected within twelve months.

### Imputation

Nineteen (31%) of the 61 trials used some form of imputation. For a description of these 19 studies see additional file 1: Description of trials with imputation of quality of life outcomes. Thirteen studies undertook imputation in the primary analysis [14-26]. Seven of these studies employed the imputation method LVCF [LOCF] [15,17,19,21,23-25]. Berry *et al*. [16] used a combination or worst value imputation and LVCF. The worse value observed in the sample was imputed if missingness was known to be due to asthma. If missingness was unrelated to asthma LVCF was used. Hseih *et al.*, [20] carried forward the baseline QoL value to the post treatment and six month follow up assessment. Buszewicz *et al*. [18] employed hotdeck imputation for missing baseline values and multiple imputation (using a predictive model) for the missing follow up scores. Kennedy *et al*. [22] imputed missing scores based on changes in other items when at least 75% of those items were present (such as IBS severity scale). Petersen *et al*. [14] used a projection method appropriate for assessing responses among subjects with neurodegenerative disease. In the remaining study, that employed imputation as part of the primary analysis, the imputation method was not specified, only that it was undertaken [26].

Six trials reported results after imputation as a sensitivity analysis. LVCF was used by four studies [27-30] and of these Peterson *et al. *[29] also considered imputing a zero value for those that were missing and McManus *et al. *[27] evaluated the use of the mean of the series. Fairbank *et al. *[31] employed multiple imputation using a regression model as part of a sensitivity analysis. Hunkeler *et al*. [32] mentioned they used imputation in a sensitivity analysis but did not specify which method.

The imputation process described above relates to missing form imputation, namely the whole QoL measure. The QoL instruments are made up of items which contribute to the score. In the case of the EQ5D measure if one of the five items is missing, the overall health status score cannot be calculated. However, in the SF36 if at least half the items in a scale are provided, the mean of the observed items is imputed for the missing items, allowing the scale score to be calculated. In the trials contained within this review, item imputation was not discussed, so it is possible this process was carried out, reducing the amount of missing data reported.

### Analysis methods

In those studies where imputation was not used (42 trials), the method of analysis was unclear in three cases (7%). A complete case analysis was undertaken in 30 of 42 trials (71%). The methods of complete-case analysis were: t-test (11 trials); analysis of variance (ANOVA) (one trial); analysis of covariance (ANCOVA) (13 trials); general linear model (one trial); Mann-Whitney test (four trials). For those studies not undertaking a complete-case analysis, a repeated measures approach was used by nine trials (22%) with eight using a linear mixed model for those patients with at least baseline data, thus allowing for some missing values in follow up data. In the ninth trial area under the curve (AUC) was used for analysis.

Following imputation 10 of 19 trials used ANCOVA, two used regression, two trials used a general linear model, two a t-test, one used generalised estimating equations, one a stratified rank test and finally one used a repeated measures model. All of the studies which employed imputation collected data for repeated assessments, but only four of the 19 trials used a repeated measures analysis.

## Discussion

This review has highlighted the need to take more account of missing data when analysing QoL outcomes in an RCT. The majority of trials identified the number of patients used in analysis by use of a flow diagram, as required by the CONSORT guidelines [13]. Half of the trials with a QoL outcome performed complete case analysis. There was no detailed discussion of the impact this had on the analysis and bias contained within the reported results. Complete case analysis is easy to perform, but has the potential to remove a large portion of the patients from the analysis. This method has two major disadvantages in that it reduces the sample size (and thus power of study) and may produce biased results unless the data are MCAR. Mixed model analysis assumes MAR data, however this assumption was not discussed by any of the authors which undertook this method of analysis.

A fifth of the articles sampled contained a QoL outcome, with nearly 31% of these performing an imputation procedure. The rationale behind the choice of imputation method was not discussed by any of articles. The review showed that of those choosing to carry out imputation, LVCF was popular. However, this method makes the assumption that the outcome is unchanged with time and in QoL situations this is unlikely. In some studies, you might carry forward an off-treatment score to an on-treatment missing value which is not likely to be that reflective the truth. As Gadbury states '...although intuitively appealing, LOCF [LVCF] requires restrictive assumptions to produce valid statistical conclusions' [5]. Carpenter and Kenward (2007) agree with this conclusion and provide a thorough critique of LOCF [LVCF] [33], warning against its use.

The results of this review support the conclusions of a review by Wood *et al.*, [34] which considered primary outcomes irrespective of type. LVCF was popular with only one trial using multiple imputation. Despite the advances in available software [12] and recommendations against LVCF [33], the researchers involved in RCTs tend to prefer this simple method.

## Conclusion

Researchers involved with the design and analysis of clinical trials should take the following into consideration. Ideally one should aim to avoid missing data at the outset though often this is impossible. The data collection method should be chosen with the aim of reducing as far as possible missing data. The reasons why data are missing should be recorded where possible.

There should be a clearer reporting of the methods used and the amount of missing data, which should be described separately for each treatment arm. The impact the missing data potentially has on results should always be discussed and a sensitivity analysis provided. Where imputation is chosen, the reason for the choice of method should be given. LVCF is usually not recommended, except in the generally implausible situation where outcome is not changing over time. Under MAR, it is desirable to include all longitudinal follow up data on the primary response, up to the end point analysis [33].

Resources that may assist researchers in deciding on appropriate action for missing data in RCTs include the recently published book by Molenberghs and Kenward (2007). The website http://www.lshtm.ac.uk/msu/missingdata/ and complementary monograph 'Missing Data in randomised controlled trials – a practical guide' [33] produced as part of an Economic and Social Research (ESRC) – Research Methods Programme. These resources will provide many helpful hints, an introduction to theory and further references that may be useful.

## Abbreviations

ANCOVA: Analysis of covariance; ANOVA: Analysis of variance; AUC: Area under the curve; BMJ: British Medical Journal; CONSORT: Consolidated Standards of Reporting Trials; ESRC: Economic and Social Research Council; GHQ: General health questionnaire; JAMA: Journal of American Medical Association; LVCF: Last value carried forward; MAR: Missing at random; MCAR: Missing completely at random; MNAR: Missing not at random; NEJM: New England Journal of Medicine; QoL: Quality of life; RCT: Randomised controlled trial; WHOQOL: World Health Organisation quality of life questionnaire.

## Competing interests

The authors declare that they have no competing interests.

## Authors' contributions

SF conceived the review, participated in data extraction, summarizing the data and drafting of the manuscript. GM, CR and JC participated in data extraction and commented on drafts of the manuscript. All authors read and approved the final manuscript.

## Supplementary Material

Additional file 1 Description of trials with imputation of quality of life outcomes. Click here for file
