# Supplementary material for: A review of RCTs in four medical journals to assess the use of imputation to overcome missing data in quality of life outcomes
Source: Trials. 2008 Aug 11;9:51. doi: 10.1186/1745-6215-9-51 (PMC3225816; doi:10.1186/1745-6215-9-51)
Supplement: Additional file 1 — Description of trials with imputation of quality of life outcomes. [file 1745-6215-9-51-S1.doc]

Additional file 1: Description of trials with imputation of quality of life outcomes

| **First**  **Author** | **Number of participants** | **Main QoL Outcomes** | **% missing at final endpoint** | **Method of Imputation** | **Imputation primary or sensitivity analysis?** | **Method of**  **Analysis** |
| --- | --- | --- | --- | --- | --- | --- |
| Ballard et al. | 82 | QoL – agitation and cognition | 14% | LVCF | Primary | ANCOVA |
| Berry *et al.* | 30 | Juniper asthma QoL scale | unclear | 1. Worst value if missing due to asthma 2. LVCF if missing not related to asthma | Primary | Paired t-test |
| Blumenthal  *et al.* | 134 | GHQ – general health  Beck depression inventory | 7% | LVCF | Primary | GLM |
| Buszewicz *et al.* | 812 | SF36 | 24% | 1. Hotdeck at baseline 2. Multiple imputation for follow up | Primary | ANCOVA |
| Fairbank  *et al.* | 349 | Oswestry disability index  SF36 | 19% | Multiple imputation | Sensitivity | ANCOVA |
| Feagan *et al.* | 181 | Inflammatory Bowel Disease Questionnaire (IBDQ) | 5% | LVCF | Primary | ANCOVA |
| Hsieh *et al.* | 129 | Roland and Morris disability questionnaire | 15% | Baseline carried forward. | Primary | ANCOVA |
| Hunkeler *et al.* | 1801 | SF12 | Not clear | Not specified | Sensitivity | t-test |
| Kaplan *et al.* | 879 | International Prostate Symptom Scores (IPSS) | 8% | LVCF | Primary | ANCOVA |
| Kennedy *et al.* | 149 | Irritable bowel syndrome symptom severity scale | 26% | Based on score changes ^*^ | Primary | Generalised Estimating Equations (GEEs) |
| Korzenik *et al.* | 124 | Inflammatory Bowel Disease Questionnaire (IBDQ) | Not clear | LVCF | Primary | Stratified rank test |
| McManus *et al.* | 441 | State anxiety inventory | 1% | 1. LVCF  2. Mean imputation | Sensitivity | GLM repeated measures |
| Meggitt *et al.* | 63 | Dermatology life quality index (DLQI) | 14% | LVCF | Sensitivity | Adjusted regression model. |
| Nair *et al.* | 144 | Health Status Questionnaire (adaption of SF36) | Not clear | LVCF | Primary | Multiple regression |
| Petersen, L. *et al.* | 547 | Global assessment of functioning and symptoms (GAF) | 32% | 1. LVCF  2. Zero value | Sensitivity | Repeated measures |
| Petersen, R. *et al.* | 769 | Alzheimer’s Disease Assessment Scale | Not clear | Projection method ^#^ | Primary | ANCOVA |
| Thomas *et al.* | 239 | SF36  Oswestry Pain disability index  McGill present pain index | 9% | LVCF | Sensitivity | ANCOVA |
| Winblad *et al.* | 248 | Alzheimer’s Disease Cooperative Study activities of daily living inventory for severe Alzheimer’s disease (ADCS-ADL-Severe) | 11% | LVCF | Primary | ANCOVA |
| Wright *et al.* | 109 | RAND Physical Function child health questionnaire | 7% | Not specified | Primary | ANCOVA |

^*^ Impute a score based on changes in other items when at least 75% of those items were present (such as IBS severity scale)

^#^ Projection method appropriate for assessing responses among subjects with neurodegenerative disease
